# High-Density Lipoproteins and the Immune System

**DOI:** 10.1155/2013/684903

**Published:** 2013-01-30

**Authors:** Hidesuke Kaji

**Affiliations:** Division of Physiology and Metabolism, University of Hyogo, 13-71 Kitaohji-cho, Akashi 673-8588, Japan

## Abstract

High-density lipoprotein (HDL) plays a major role in vasodilation and in the reduction of low-density lipoprotein (LDL) oxidation, inflammation, apoptosis, thrombosis, and infection; however, HDL is now less functional in these roles under certain conditions. This paper focuses on HDL, its anti-inflammation behavior, and the mechanisms by which HDL interacts with components of the innate and adaptive immune systems. Genome-wide association studies (GWAS) and proteomic studies have elucidated important molecules involved in the interaction between HDL and the immune system. An understanding of these mechanisms is expected to be useful for the prevention and treatment of chronic inflammation due to metabolic syndrome, atherosclerosis, or various autoimmune diseases.

## 1. Introduction 

**Figure 1:**
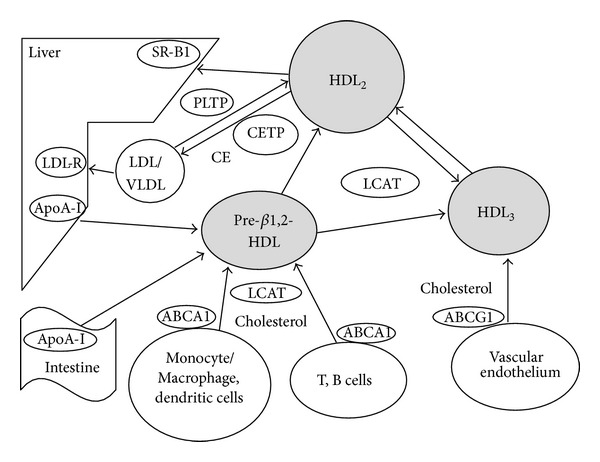
Dynamics of HDL particles and immune cells. SR-BI: scavenger receptor type B1, LDL-R: LDL receptor, CETP: cholesterol ester transfer protein, PLTP: phospholipid transfer protein, LCAT: lecithin:cholesterol acyltransferase, ABCA1: ATP binding cassette transporter A1, ABCG1: ATP binding cassette transporter G1.

High-density lipoprotein (HDL) contains free or esterified cholesterol, phospholipids, triglycerides, and various proteins, including apolipoproteins, enzymes, and transfer proteins. The most abundant HDL apolipoproteins are apoA-I and apoA-II; less abundant are apoC, apoE, apoD, and apoJ. HDL enzymes include lecithin:cholesterol acyltransferase (LCAT), serum paraoxonase-1 (PON1) [1–3], and platelet-activating factor acetylhydrolase (PAF-AH) [4]. Transfer proteins include cholesteryl ester transfer protein (CETP) and phospholipid transfer protein (PLTP). Furthermore, chromatography and mass spectrometry have revealed many other proteins in HDL [5, 6]. HDL particles can be subclassified into small discoidal HDL (pre-*β*_1_ HDL and pre-*β*_2_ HDL), intermediate spherical HDL_3_ (HDL_3c_, HDL_3b_, and HDL_3a_), and large, cholesterol-rich spherical HDL_2_ (HDL_2a_ and HDL_2b_) [7–10] (Figure 1). Large HDL_2_ particles interact with liver scavenger receptors class B type 1 (SR-B1), which ensures the delivery of cholesterol to the liver [11]. Intermediate HDL_3_ induces cholesterol efflux through the ATP-binding cassette transporter G1 (ABCG1) [12]. Small HDL particles promote cholesterol efflux through the ATP-binding cassette transporter A1 (ABCA1) [13]. Accumulating evidence suggests that in addition to reverse transport of cholesterol from the periphery to the liver, HDL plays a major role in vasodilation and in the reduction of LDL oxidation [14], inflammation, apoptosis, thrombosis, and infection [15]. During infection, both innate and adaptive immunities are involved in the inflammatory process and the immune response. Innate immunity is a nonspecific defense mechanism comprising cellular and humoral responses. The cellular response includes antigen-presenting cells such as macrophage and dendritic cells. The humoral response includes various effectors, such as the complement cascade or soluble pattern recognition receptors (PRRs). Adaptive immunity is an antigen-specific defense mechanism against foreign antigens or pathogens. The principal effectors of adaptive immunity are B lymphocytes (humoral response) and T lymphocytes (cellular response). This paper focuses on the role of HDL in the immune system [16, 17] and in the pathogenesis of atherosclerosis and other types of immune-mediated disease. 

## 2. HDL and Innate Immunity 

Innate immunity is an ancient defense mechanism that humans inherited from invertebrates and that they use against a variety of pathogens. The main cells involved in innate immunity are monocyte-derived macrophage and dendritic precursor cells. Additional cells include natural killer cells, neutrophiles, eosinophiles, mast cells, basophiles, and epithelial cells. These cells use PRRs to recognize pathogen-associated molecular patterns (PAMPs). These PRRs include c-type lectins, leucin-rich proteins, macrophage scavenger receptors, pentraxins, lipid transferase, integrins, and inflammasome proteins [18, 19]. PAMP recognition leads to activation and production of the complement cascade, cytokines, and antimicrobial peptides [20]. In addition, PAMPs stimulate the differentiation of dendritic precursors into antigen-presenting, mature dendritic cells and trigger the adaptive immune system [20]. 

### 2.1. Acute Phase

During the acute phase of inflammation, mediators such as tumor necrosis factor-*α* (TNF-*α*) and interleukin-6 (IL-6) induce serum amyloid A (SAA) and group IIA secretary phospholipase A (sPLA_2_-IIA), which markedly change the composition of HDL apolipoproteins and lipids [21, 22]. ApoA-1 gene expression and plasma half-life decrease [23, 24]. SAA rapidly becomes the most abundant protein in association with HDL [25]. PON1 enzyme activity decreases and, thereby, the antioxidant properties of HDL are reduced [26]. PAF-AH is increased, thus leading to increased levels of proatherogenic lipids [27, 28]. The altered composition of HDL lipids includes decreased levels of cholesteryl ester and phospholipids and increased levels of triglycerides, free cholesterol, ceramides, and glucosylceramides [29]. 

Acute phase HDL is associated with disease activity; a decreased number of small HDL particles is inversely associated with the disease activity score and C-reactive protein (CRP) level [30]. 

### 2.2. Protection from Sepsis

Lipopolysaccharide (LPS) is the primary cause of sepsis induced by gram-negative bacteria. LPS, LPS-binding protein, CD14, and the toll-like receptor 4 (TLR4) complex induce macrophage activation [31]. HDL, particularly apoA-I, decreases macrophage activation by binding and neutralizing LPS [32]. The HDL receptor in the liver, SR-B1 also provides important protection against sepsis [33, 34]. SR-B1 deficiency results in a reduced rate of survival following sepsis [33]. SR-B1 also modulates TLR4 signaling in macrophages and helps facilitate LPS removal from circulation [33, 34]. HDL_2_ modulates SR-B1 function by reverse transporting core cholesteryl ester to the liver via SR-B1, enabling the production of pre-*β* HDL which effectively removes cholesterol from macrophages, dendritic cells, and lymphocytes. 

In clinical sepsis, a positive correlation is evident between PLTP activity and acute-phase markers such as CRP and LPS-binding proteins. During human experimental endotoxemia, PLTP activity decreases at the time of LPS infusion and transiently increases during reconstituted HDL infusion. PLTP can accelerate the disturbance of lipoprotein homeostasis, thereby playing a role in the attenuation of the acute-phase response [35]. 

### 2.3. Cellular Innate Response

**Figure 2:**
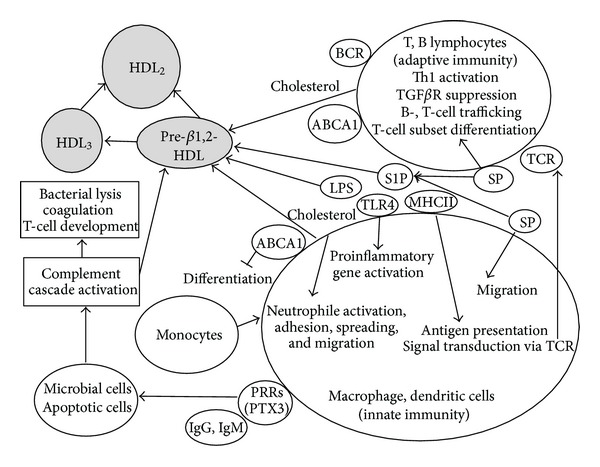
HDL and innate as well as adaptive immune cell functions. LPS: lipopolysaccharide, TLR4: Toll-like receptor 4, MHC II: major histocompatibility complex class II, SP: sphingolipid, S1P: sphingosine-1-phosphate, PRRs: pattern recognition receptors, PTX3: pentraxin 3, TCR: T-cell receptor, BCR; B-cell receptor.

Macrophages and dendritic cells are antigen-presenting cells that are crucial to innate immunity. The cell surfaces of macrophage and dendritic cells express costimulatory molecules, which are required for stimulation of the adaptive cellular immune system, and lipid rafts, which are microdomains that contain high concentrations of cholesterol, sphingolipids, and proteins integral to signaling, protein transport, and adhesion [36, 37]. The shifting composition of lipid rafts particularly decreases in cholesterol, downregulates some cellular functions [38], including the activation, adhesion, spread, and migration of neutrophiles. HDL, or particularly apoA-I, is involved in interaction with ABCA1 or ABCG1 and removes cholesterol from the lipid rafts in macrophages and dendritic cells [39, 40] (Figure 2). Thus, HDL negatively regulates T-cell activation and the expression of inflammatory mediators in macrophages and dendritic cells. In macrophages, T-cell inactivation is caused by decreased macrophage expression of major histocompatibility complex class II (MHC II), which is a lipid raft component critical to antigen presentation [41–43]. ApoA-I of HDL inhibits the differentiation of monocytes to dendritic cells by increasing monocyte secretion of prostaglandin E2 (PGE2) and IL-10 [44]. It also inhibits T-lymphocyte activation by decreasing antigen presentation in differentiated dendritic cells [45]. 

Receptors from the TLR family are expressed on the surface of macrophages and dendritic cells. TLRs are involved in the innate immune response to infections. In rodent and human atherosclerotic lesions, TLRs, particularly TLR1, TLR2, and TLR4, play a role in T-lymphocyte activation by recruiting and activating leucocytes, regulating foam cell formation, and controlling antigen presentation [46, 47]. Some phospholipids in HDL function directly in immunoregulation by modulating dendritic cells for their ability to activate T helper type 1 (Th1) cells [48]. A well-characterized TLR ligand, LPS, upregulates a large number of proinflammatory genes in macrophages. Through TLR4 interaction, HDL inhibits LPS-induced antiviral response in macrophages [49, 50]. Lipid raft integrity is crucial to LPS-induced monocyte activation. ApoA-I and its mimetic peptide deplete cholesterol from lipid rafts of monocytes and thereby reduce TLR4 expression [51]. 

The other major class of lipid rafts is sphingolipids, which are metabolized to ceramide and subsequently to sphingosine, a metabolite that becomes phosphorylated by sphingosine kinase (SPHK) to generate sphingosine-1-phosphate (S1P) [52]. The S1P receptor 2 (SIP2) inhibits macrophage migration. Free or albumin-bound S1P rapidly degrades in most tissues, but HDL-bound S1P is less susceptible to degradation [53]. The mechanism by which HDL removes S1P from lipid rafts remains unclear but may involve specific molecules such as ABCA1. HDL-bound S1P is enriched with small, dense HDL_3_ and positively correlates with serum levels of HDL cholesterol, apoA-I, and apoA-II [52]. The central role of S1P and SPHK in the pathogenesis of several inflammatory disorders, including rheumatoid arthritis (RA), asthma, and atherosclerosis, is well known [54]; however, additional studies are required to clarify the role of HDL-bound S1P. 

### 2.4. Humoral Innate Response

Innate immunity consists of a highly regulated immune surveillance system comprising several humoral factors, including soluble PRRs, such as collectins, ficolins, and pentraxins [32, 33], and the complement cascade [34]. PRRs, IgG, and IgM clusters recognize microbial or apoptotic cells and activate the complement cascade, which leads to the assembly of a terminal complement complex, bacterial lysis, and activation of several nonlethal signals that promote opsonization, chemotaxis, and TLR signaling [34] (Figure 2). The complement cascade coordinates innate defenses and potentiates coagulation to provide a mechanical barrier against bacterial spread. Activation of the complement also modulates antigen-presenting cells, macrophages, and dendritic cells, resulting in the regulation of T-lymphocyte development. Recent proteomic analyses in healthy subjects [5, 6] revealed several types of HDL particles, including complement components C4a, C4b, C9, and vitronectin. In contrast, HDL particles detected in patients with coronary artery disease include complement C3 [5]. In vitro experiments on endothelial cells have shown that HDL inhibits the formation of the terminal attack complex of the complement [55, 56]. Another study has shown that plasma HDL levels inversely correlate with terminal complex C5b–C9 levels [57]. This evidence suggests that HDL binds complements and enhances complement clearance. 

A member of the pentraxin subfamily, PTX3, is soluble PRR. PTX3 deficiency leads to invasive pulmonary aspergillosis due to the defective recognition of conidia by alveolar macrophages and dendritic cells. PTX3 deficiency also causes inappropriate induction of an adaptive type 2 response [58] and some types of cardiovascular disease, including atherosclerosis [59, 60]. HDL induces mRNA expression and protein release of PTX3. This HDL effect is dependent on lysosphingolipid receptors, the PI3K/Akt axis, and is mimicked by S1P [61]. PTX mRNA increase in the aorta of transgenic mice that overexpress human apoA-I, whereas PTX mRNA decreasess in the aorta of apoAI knockout mice. HDL injection results in increase in plasma PTX3 levels in C57BL/6 mice [61]. Thus, the anti-inflammatory mechanism of HDL likely involves PTX3 activation. 

## 3. HDL and Adaptive Immunity

The adaptive immune system is found only in vertebrates and is characterized by antigen-specific responses to pathogens. The principle components of adaptive humoral immunity are B lymphocytes that originate in the bone marrow. The principal components of cellular immunity are T lymphocytes that originate from hematopoietic cells and mature in the thymus. Gene rearrangement generates the antigen-specific receptors expressed in lipid rafts on the surface of T or B cells. Therefore, T and B lymphocytes incorporate specificity and immune memory in vertebrate host defenses. 

The key receptor in B cells is the B-cell receptor (BCR), and the key receptor of T cells is the T-cell receptor (TCR). BCR and TCR are located in lipid rafts. Removal of cholesterol from BCR lipid rafts by HDL affects several modes of B-cell activation, including BCR-initiated signal transduction, endocytosis of BCR-antigen complexes, loading of antigenic peptides onto MHC-II, MHC-II-associated antigen presentation to T cells, and detection of helper signals via the CD40 receptor [62]. The HDL-induced cholesterol efflux from macrophages also affects antigen presentation to T cells as well as TCR signaling [63–65] (Figure 2). 

S1P regulates B- and T-cell trafficking as well as differentiation of T cell subsets. S1P inhibits forkhead box P3 (FoxP3)^+^ in regulatory T cells (Tregs) but stimulates the development of Th1 cells [65]. S1P controls the dichotomy between these two T-cell lineages by antagonizing transforming growth factor *β* (TGF-*β*) [66]. ApoA-I suppresses inflammation by stimulating Tregs in the lymph nodes and by inhibiting effectors such as memory T cells [67]. 

## 4. HDL and Immune-Mediated Disease

### 4.1. Autoimmune Disease

Plasma HDL cholesterol (HDL-C) levels are elevated in multiple sclerosis and reduced in autoimmune diseases such as systemic lupus erythematosus (SLE), RA [68], Sjögren's syndrome [69], ankylosing spondylitis [70], psoriatic arthritis, and inflammatory bowel disease [71]. Proinflammatory HDL is detected in 45% of SLE patients and 20% of RA patients [72]. Relative to HDL, proinflammatory HDL is less capable of reverse cholesterol transport, antioxidation and other anti-inflammatory roles because it contains lower levels of apoA-1 and higher levels of monocyte chemoattractant protein-1 (MCP-1) and cellular adhesion molecules such as intercellular adhesion molecule-1 (ICAM-1) and vascular cell adhesion molecule-1 (VCAM-1) [73]. Thus, it is important to quantify HDL-C and measure HDL quality. The representative methods used to measure HDL quality are the monocyte chemotaxis assay or the cell-free assay developed by Navab et al. [74, 75]. In addition to SLE and RA, factors that promote proinflammatory HDL include coronary atherosclerosis, diabetes mellitus, hemodialysis, a high saturated fat diet, infection, and surgery [73]. 

 In SLE and RA, B and T cells are the main components of pathogenesis. In SLE, antibodies against apoA-I or HDL are associated with persistent disease activity [76]. In RA patients, the oxidative LDL antibody correlates positively with CRP and negatively with plasma HDL [77]. 

### 4.2. Metabolic Disease and Atherosclerosis

Genome-wide association studies (GWAS) using high throughput techniques have uncovered significant genetic variation in association with plasma HDL-C levels [78–81]. Among these genetic variations, CETP, lipoprotein lipase (LPL), ABCA1, hepatic lipase (LIPC), and endothelial lipase (LIPG) exhibit highly significant associations with plasma HDL-C levels. Newly identified loci, including GALNT2, are associated with plasma HDL-C levels. 

A reduced plasma HDL-C level is a factor of metabolic syndrome, which causes obesity and chronic inflammation [82, 83]. We have previously shown that two single-nucleotide polymorphisms (SNPs) of the promoter region of the neuropeptide Y (NPY) receptor Y2 gene are associated with altered levels of plasma HDL-C [84]. Kuo et al. reported that stress plus a diet high in fat and sugar cause increased NPY secretion from the sympathetic nerve terminal and thereby trigger metabolic syndrome through the NPY receptor Y2 [85]. It is interesting to speculate whether the SNPs of the NPY receptor Y2 affect HDL-C expression levels directly or plasma HDL-C expression indirectly through certain molecules such as CETP, LPL, LIPC, and apoA-1 in the liver; ABCA1 in monocyte/macrophages and dendritic cells; or LIPG, ABCG1, and LPL in the endothelium. Furthermore, in obese subjects, a number of metabolic and immune genes that exhibited expression in subcutaneous adipose tissues correlating with plasma HDL-C levels were identified [86]. Reduced levels of plasma HDL-C are one of the risk factors for atherosclerosis-induced cardiovascular events. Treg cells play an important role in adaptive immunity and become elevated in acute myocardial infarction. A study demonstrated significant inverse correlations between levels of Treg cells and plasma HDL-C [87]. HDL_3_ induces in vitro and in vivo anti-inflammatory signals such as TGF-*β*_2_ expression in endothelial cells or various signals in transgenic mice overexpressing human apoA-1 and apoA-1 knockout mice [88]. 

PLTP is a protein involved in HDL remodeling. Vergeer et al. reported that 2 PLTP SNPs are associated with lower PLTP transcription and activity, an increased number of HDL particles, smaller HDL size, and decreased risk of cardiovascular disease [89]. 

 Carriers with a functional mutation in SR-B1 yield higher plasma levels of HDL-C and reduced efflux of cholesterol from macrophages, but no significant increases in atherosclerosis [90]. Reduced SR-B1 function associates with altered platelet function and decreased adrenal steroidogenesis [89]. Low levels of plasma HDL as a result of heterozygosity for loss-of-function mutations in ABCA1 do not associate with an increased prevalence of ischemic heart disease [91].

Tangier disease is a genetic disorder that results from ABCA1 deficiency and results in extremely low levels of HDL-C and premature atherosclerosis. The immunological features of this disease are not well defined. The results of an in vitro study on Tangier fibroblasts indicate that ABCA1 forms a complex with syntaxin 13 and flotillin-1, which resides the plasma membrane and phagosomes partially located in raft microdomains [92]. 

In contrast, CETP deficiency is a genetic disorder that results in extremely high levels of HDL-C. However, the long life span of these patients is not still evident. In accordance with this evidence, recent randomized prospective study resulted that CETP inhibitor dalcetrapib increased HDL-C levels but did not reduce the risk of recurrent cardiovascular events in patient who had had a recent acute coronary syndrome [93]. 

## 5. Conclusions 

Accumulating evidence suggests that HDL or a specific apolipoprotein associated with HDL, such as apoA-I, is involved in the innate and adaptive immune responses primarily through the modulation of lipid raft components in monocytes/macrophages, dendritic cells, and T and B lymphocytes. Plasma HDL-C is usually reduced in chronic inflammation. These findings suggest that HDL protect against inflammation. However, chronic inflammation modifies HDL from a molecule with anti-inflammatory properties to one with proinflammatory properties, which leads to complex interpretation of plasma HDL-C levels. Although recent genetic and proteomic studies have unveiled important molecular players in HDL metabolism and immune activity, the mechanism for HDL regulation by these molecules remains unclear. Additional studies are required to answer several questions about HDL-C and inflammatory disease with regard to reduced plasma HDL-C levels as potential pathogenic cause of inflammatory diseases; HDL-C consumption and its consequences versus benefits for protection against these diseases; and altered HDL function in these diseases.
